# Alternative protocol to initiate high-frequency oscillatory ventilation: an experimental study

**DOI:** 10.1186/cc5052

**Published:** 2006-09-25

**Authors:** Jens Karmrodt, Matthias David, Shying Yuan, Klaus Markstaller

**Affiliations:** 1 Department of Anesthesiology, Johannes Gutenberg-University, Langenbeckstraße 1, D-55101 Mainz, Germany

## Abstract

**Introduction:**

The objective was to study the effects of a novel lung volume optimization procedure (LVOP) using high-frequency oscillatory ventilation (HFOV) upon gas exchange, the transpulmonary pressure (TPP), and hemodynamics in a porcine model of surfactant depletion.

**Methods:**

With institutional review board approval, the hemodynamics, blood gas analysis, TPP, and pulmonary shunt fraction were obtained in six anesthetized pigs before and after saline lung lavage. Measurements were acquired during pressure-controlled ventilation (PCV) prior to and after lung damage, and during a LVOP with HFOV. The LVOP comprised a recruitment maneuver with a continuous distending pressure (CDP) of 45 mbar for 2.5 minutes, and a stepwise decrease of the CDP (5 mbar every 5 minute) from 45 to 20 mbar. The TPP level was identified during the decrease in CDP, which assured a change of the P_a_O_2_/F_I_O_2 _ratio < 25% compared with maximum lung recruitment at CDP of 45 mbar (CDP45). Data are presented as the median (25th–75th percentile); differences between measurements are determined by Friedman repeated-measures analysis on ranks and multiple comparisons (Tukey's test). The level of significance was set at *P *< 0.05.

**Results:**

The PaO_2_/FiO_2 _ratio increased from 99.1 (56.2–128) Torr at PCV post-lavage to 621 (619.4–660.3) Torr at CDP45 (CDP45) (*P *< 0.031). The pulmonary shunt fraction decreased from 51.8% (49–55%) at PCV post-lavage to 1.03% (0.4–3%) at CDP45 (*P *< 0.05). The cardiac output and stroke volume decreased at CDP45 (*P *< 0.05) compared with PCV, whereas the heart rate, mean arterial pressure, and intrathoracic blood volume remained unchanged. A TPP of 25.5 (17–32) mbar was required to preserve a difference in P_a_O_2_/F_I_O_2 _ratio < 25% related to CDP45; this TPP was achieved at a CDP of 35 (25–40) mbar.

**Conclusion:**

This HFOV protocol is easy to perform, and allows a fast determination of an adequate TPP level that preserves oxygenation. Systemic hemodynamics, as a measure of safety, showed no relevant deterioration throughout the procedure.

## Introduction

Current ventilatory strategies for 'lung-protective' ventilation in acute respiratory distress syndrome (ARDS) include low tidal volumes to avoid alveolar overdistension, adequate end-expiratory lung volume by positive end-expiratory pressure to prevent end-expiratory alveolar collapse, and inspiratory pressure limitation to minimize further stress and strain to the lung fibrous skeleton [1]. The excessive and nonphysiological strain to lung structures is caused by high transpulmonary pressures (TPP), which in turn depend on the respiratory system elastance [2]. High-frequency oscillatory ventilation (HFOV) offers several advantages over conventional ventilation. Oscillations in HFOV are superimposed on a constant fresh gas flow and induce active inspiratory and expiratory gas movement, resulting in high constant mean airway pressures at low tidal volumes. Atelectatic lung regions are reopened by the continuous distending airway pressure (CDP), and the superimposed small oscillations provide alveolar gas exchange for CO_2 _removal [3]. Recruitment maneuvers are beneficial at initiation of HFOV to ensure sufficient gas exchange area in the diseased lung [4].

In most clinical studies, HFOV is initiated by an initial lung volume optimization procedure (LVOP) with a CDP level 5 mbar above the effective mean airway pressure previously used at conventional ventilation [5-8]. The CDP is thereafter increased in a stepwise manner (2–5 mbar every 15–30 minutes) up to the maximum increase of PaO_2 _or up to a predetermined CDP. This maneuver is followed by a stepwise reduction of the CDP (2 mbar every 30 minutes up to 4 hours) to maintain alveolar patency. Recruitment in a stepwise fashion is effective and safe with regard to hemodynamic impairment, but is also time consuming in adjusting an effective CDP.

Preclinical and clinical trials have been presented recently that used a recruitment maneuver with a high CDP, followed by a stepwise decrease of the airway pressure. Sedeek and colleagues used a continuous positive airway pressure of 50 mbar for lung recruitment in lung-lavaged sheep. The CDP was then set according to the maximal compliance on the pressure-volume [9]. The Treatment with Oscillation and an Open Lung Strategy (TOOLS) trial used a standardized HFOV protocol in patients, which showed that the combination of HFOV and a high initial recruitment maneuver (with interrupted HFOV) resulted in a rapid and sustained improvement in oxygenation. The mean airway pressure was then titrated in a decremental fashion according to the oxygenation response [10].

In the present study we investigated the immediate effect of a modified LVOP by means of ongoing HFOV on hemodynamics and oxygenation prior to its clinical application. To demonstrate the feasibility and safety of this lung optimization procedure, HFOV was initiated with the CDP set to 45 mbar (for 2.5 minutes) during ongoing oscillation in six pigs after saline lung lavage, simulating an early-phase ARDS model. The CDP was thereafter reduced in a stepwise fashion of five mbar every five minutes with simultaneous measurement of the TPP. The effects upon gas exchange, systemic hemodynamics, and the pulmonary shunt proportion were observed at each CDP and TPP level.

## Methods

### Animal preparation

The study protocol was approved by the institutional and state animal care committee. Six pigs were anesthetized after premedication (8 mg/kg azaperone and 0.02 mg/kg atropine intramuscularly) with 0.01 mg/kg fentanyl (Fentanyl; Janssen Pharmaceuticals, Neuss, Germany) and 5 mg/kg thiopentone (Trapanal; Altana Pharma, Konstanz, Germany) intravenously. Anesthesia was maintained by continuous infusion of thiopentone (6–9 mg/kg/hour) and fentanyl (5 μg/kg/hour). The trachea was intubated by tracheotomy (endotracheal tube, ID 9 mm; Ruesch, Kernen, Germany) and the pigs were ventilated in a pressure-controlled mode (PCV) with a FiO_2 _of 0.3 in air, a positive end-expiratory pressure of 5 mbar, and a variable respiratory rate to achieve an end-tidal carbon dioxide tension of 40 ± 5 Torr (Servo 900 C; Siemens, Erlangen, Germany). Ringer's solution was substituted continuously at a rate of 5 ml/kg/hour intravenously.

Instrumentation included arterial and central venous catheterization by femoral cutdown for blood pressure monitoring (S/5 Monitoring; Datex-Ohmeda, Duisburg, Germany), blood gas analysis, and drug administration. A pulmonary arterial catheter was introduced via the right internal jugular vein for mixed venous blood gas sampling (Radiometer 500 and OSM 3; Radiometer, Copenhagen, Denmark). A left ventricular catheter was introduced through the right internal carotid artery for measurement of the left ventricular end-diastolic pressure (LVEDP). The position of all catheters was verified by transduction of typical pressure waveforms and was verified by autopsy after the experiment. All intravascular pressures were referenced to the mid-chest level. For measurement of the esophageal pressure, a catheter with an inflatable balloon on its tip (Oesophagus Catheter; Jaeger GmbH, Hoechberg, Germany) was placed in the distal esophagus and filled with 1 ml air [11]. A pneumotachymeter (Pneumotachymeter; Jaeger-Toennies, Hoechberg, Germany) was attached to the endotracheal tube. The airway and esophageal pressures were recorded and analyzed with a dedicated monitoring system (MasterScreenIOS; Jaeger-Toennies). After finishing the study protocol, the animals were euthanized (40 mval potassium chloride intravenously) in deep anesthesia.

### Lung lavage model

A surfactant-depletion model was induced by repetitive lung lavages (4 ± 1) until a PaO_2_/FiO_2 _ratio less than 100 Torr was achieved. Isotonic Ringer's solution (20 ml/kg, 38°C) was instilled into the endotracheal tube, and the fluid was retrieved by gravity drainage after 30 seconds of apnoea. To maintain hemodynamic stability, a continuous infusion (mean ± standard deviation) of 3 ± 2 μg/kg/hour epinephrine was administered during the lung lavages and was kept constant during the entire study protocol. After lung lavages, the animals were ventilated with a positive end-expiratory pressure of 5 mbar during PCV (inspiratory pressure = 25 mbar, FiO_2 _= 1.0, inspiration time:expiration time ratio = 1:1) for 120 minutes prior to initiation of HFOV.

### High-frequency oscillatory ventilation

**Figure 1:**
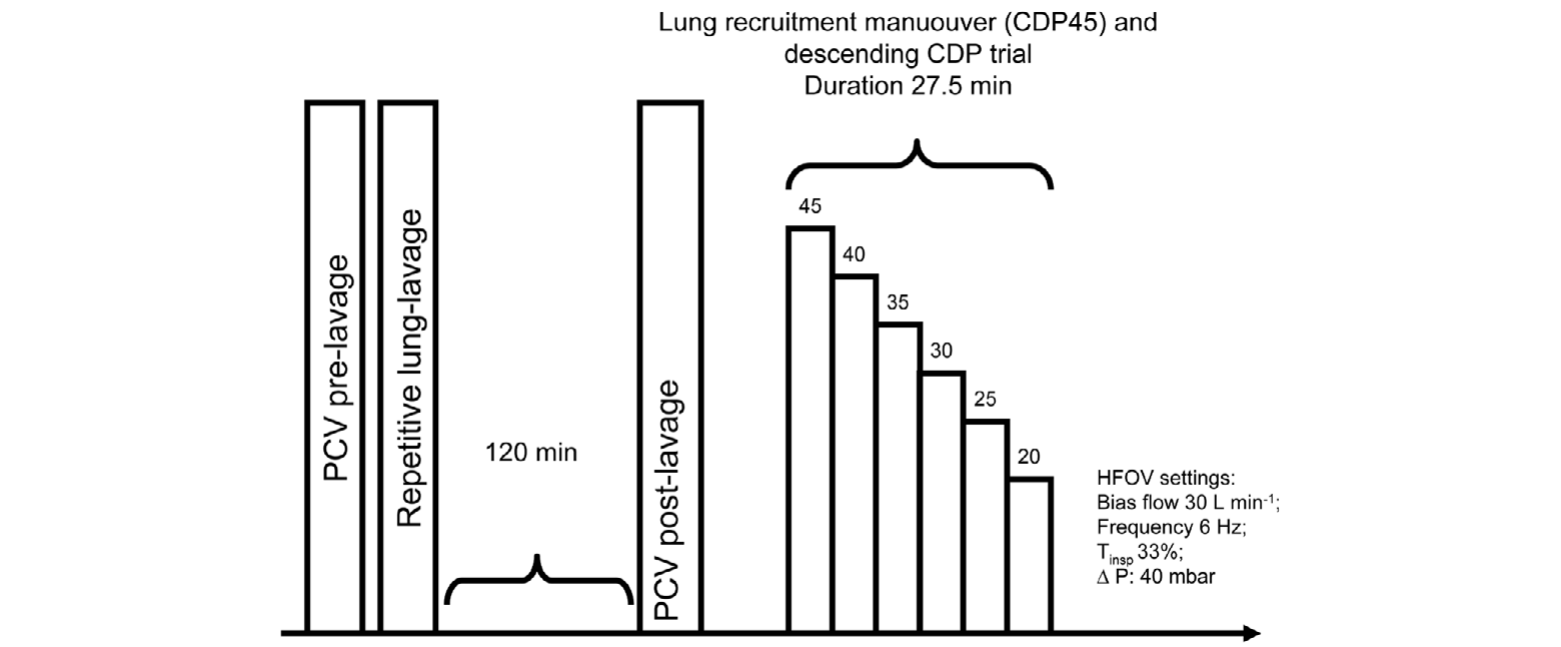
Illustration of the time course of the study protocol. CDP, continuous distending pressure; CDP45, continuous distending pressure of 45 mbar; ΔP, pressure amplitude; HFOV, high-frequency oscillatory ventilation; PCV, pressure-controlled ventilation; T_insp_, inspiration time.

A commercially available HFOV oscillator (Sensormedics 3100 B; Yorba Linda, California, USA) was used. Hemodynamic stability before HFOV initiation was defined by a mean arterial pressure (MAP) > 60 mmHg and a pulmonary artery occlusion pressure (PAOP) > 10 mmHg. If the PAOP was inadequate, repetitive boluses of 5 ml/kg colloids within 10 minutes were applied until hemodynamic stability was achieved. The following HFOV settings were used and kept constant throughout the entire protocol (Figure 1): FiO_2 _of 1.0, an oscillatory frequency of 6 Hz, an inspiration time of 33% of the respiratory cycle, a bias flow of 30 l/minute, and a pressure amplitude of 40 mbar.

The protocol for lung volume optimization during HFOV comprised three steps: step 1, a lung recruitment maneuver – setting the initial CDP during ongoing oscillations to 45 mbar (CDP45) for 2.5 minutes; step 2, decrease of the CDP – the CDP was reduced in a stepwise manner by 5 mbar every 5 minutes from 45 mbar to 40 mbar, 35 mbar, 30 mbar, 25 mbar, and 20 mbar (CDP20) with simultaneous measurement of the TPP; and step 3, identification of the optimal TPP – the TPP level necessary to maintain lung recruitment was defined as the TPP necessary to prevent a decrease in the PaO_2_/FiO_2 _ratio > 25% compared with the PaO_2_/FiO_2 _ratio at CDP45 (that is to say, maximum lung recruitment).

### Measurements

The following parameters were recorded before and 120 minutes after initiation of lung damage during PCV, and at every CDP level during HFOV: the heart rate, the MAP, the right atrial pressure, the PAOP, the mean pulmonary artery pressure, and the LVEDP. In addition, the intrathoracic blood volume, the extravascular lung water, and the cardiac output (CO), as obtained by the PiCCO^®^-Technology system (Pulsion Medical Systems, Munich, Germany), were recorded. For blood gas analyses, arterial and mixed venous blood samples were drawn (ABL 500/OSM 3; Radiometer, Copenhagen, Denmark). The pulmonary vascular resistance, the systemic arterial vascular resistance, the stroke volume (SV), oxygen delivery, the oxygenation index, and the pulmonary shunt proportion were calculated according to standard formula.

The TPP was calculated as the difference between the CDP (measured at the proximal end of endotracheal tube) and the esophageal pressure.

### Statistical analysis

Data are expressed as the median, and 25th and 75th percentiles (interquartile range). Intraindividual differences before and after induction of lung injury and during the recruitment maneuver (PCV post-lavage and CDP45) were tested nonparametrically using the Wilcoxon signed-rank test. Any differences during the fast CDP deceleration trial were addressed by a Friedman repeated-measures analysis of variance on ranks and multiple comparisons by Tukey's test. *P *< 0.05 was considered significant (SigmaStat Version 2.03; SPSS Inc., San Raphael, California, USA).

## Results

All six animals (25 ± 2 kg bodyweight, mean ± standard deviation) completed the entire study protocol. Hemodynamic and gas exchange variables before and after induction of lung damage are presented in Table 1. The MAP and PAOP before initiation of HFOV complied with the predefined requirements; that is, PAOP of 13 (11–13) mmHg and MAP of 83 (82–85) mmHg. Repetitive lung lavages decreased the PaO_2_/FiO_2 _ratio (PCV pre-lavage, 559 (535–658) Torr vs PCV post-lavage, 99 (56–128) Torr; *P *< 0.05), and increased the pulmonary shunt fraction (PCV pre-lavage, 9.97% (8.8–11%) vs PCV post-lavage, 51.8% (49–55%); *P *< 0.05). The oxygenation index increased from PCV pre-lavage (1.4 (1.2–2)) to PCV post-lavage (16.3 (14.6–21.3)) (*P *< 0.05). The extravascular lung water increased from PCV pre-lavage (290 (241–311) ml) to PCV post-lavage (420 (354–463) ml) (*P *< 0.05).

### Hemodynamics

Hemodynamic variables are presented in Table 1 for the lung recruitment maneuver and in Table 2 for the stepwise decrease of the CDP. The MAP, heart rate and intrathoracic blood volume did not change throughout the entire experiment. The right atrial pressure, mean pulmonary artery pressure, PAOP, and LVEDP during the lung recruitment procedure increased significantly from the PCV post-lavage to CDP45 (*P *< 0.05). The CO and SV decreased from PCV post-lavage (CO, 3.6 (3.1–3.9) l/minute; SV, 32 (31–35) ml) to CDP45 (CO, 2.6 (2.3–3.1) l/minute; SV, 19 (18–24) ml) (*P *= 0.031).

### Pulmonary gas exchange and pulmonary shunt fraction

**Figure 2:**
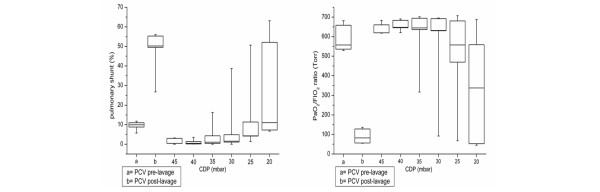
PaO_2_/FiO_2 _ratio and shunt fraction during pressure-controlled ventilation and high-frequency oscillatory ventilation. The PaO_2_/FiO_2 _ratio and shunt fraction during pressure-controlled ventilation (PCV) pre-lavage and PCV post-lavage in relation to continuous distending pressure (mbar). CDP, continuous distending pressure.

The LVOP increased the PaO_2_/FiO_2 _ratio from PCV post-lavage (99 (56–128) Torr) to CDP45 (621 (619–660) Torr) (*P *< 0.05). The pulmonary shunt fraction decreased from PCV post-lavage (51.8% (49–55%)) to CDP45 (1.03% (0.4–3%)) (*P *< 0.05). During the stepwise decrease of the CDP, the shunt fraction increased to 20.2% (7.2–52%) at CDP20 compared with 1.03% (0.4–1.4%) at CDP45 and compared with 0.7% (0.1–1.4%) at CDP of 40 mbar (*P *< 0.05). The PaO_2_/FiO_2 _ratio decreased from CDP45 (621 (619–660) Torr) to CDP20 (429 (52–558) Torr) (*P *< 0.05) (Figure 2). The PaCO_2 _increased from CDP45 (38.5 (28.3–46) Torr) to CDP20 (54.4 (40.7–68.6) Torr) (*P *< 0.001).

### Identification of the required TPP level

The lung optimization procedure in this study required less than 30 minutes to recruit the lung and to identify the lowest TPP level to maintain adequate oxygenation and gas exchange. The TPP increased from PCV post-lavage (10 (8–11) mbar) to CDP45 (36 (26–43) mbar) (*P *< 0.05).

**Figure 3:**
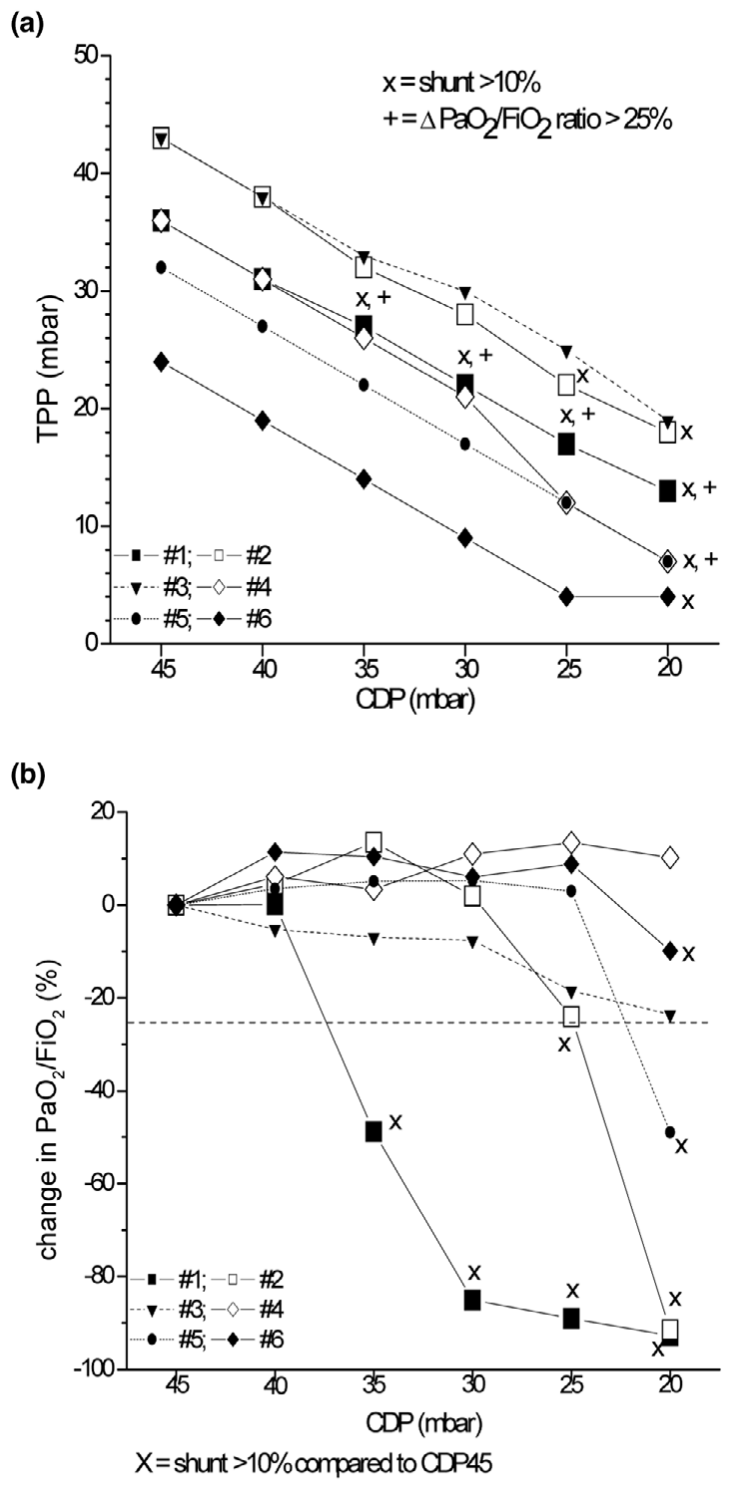
Relationship between continuous distending pressure and transpulmonary pressure or changes in PaO_2_/FiO_2 _ratio. Relationship (for each animal) between the continuous distending pressure (CDP) (mbar) and: (a) the transpulmonary pressure (TPP) (mbar) (X, shunt fraction > 10%; +, decrease of the PaO_2_/FiO_2 _ratio > 25%), and (b) changes in the PaO_2_/FiO_2 _ratio (%) (dotted line, decrease of the PaO_2_/FiO_2 _ratio of 25% compared with CDP of 45 mbar (CDP45); X, shunt fraction > 10%).

A TPP between 32 and 14 mbar prevented a decrease in the PaO_2_/FiO_2 _ratio > 25% compared with the PaO_2_/FiO_2 _ratio at CDP45. These TPP levels were achieved at CDP settings ranging from 25 to 40 mbar (Figure 3a). In three animals the PaO_2_/FiO_2 _ratio did not decrease more than 25% compared with the measurement at CDP45, independently of the applied CDP (Figure 3b). On average, a TPP of 25.5 (17–32) mbar was required to preserve a difference in the PaO_2_/FiO_2 _ratio < 25% related to CDP45; this TPP was achieved at a CDP of 35 (25–40) mbar.

## Discussion

The present experimental study investigated the effects upon gas exchange, TPPs, and hemodynamics of a modified HFOV initiation protocol to optimize the lung volume in a porcine model of acute lung injury. The protocol consists of a fast recruitment maneuver followed by a stepwise decrease of CDP. The fast stepwise reduction of CDP allows the identification of the lowest TPP level required to maintain improvement of oxygenation in each animal. This approach therefore offers an effective reduction of pulmonary shunt fraction and improvement in oxygenation without relevant adverse hemodynamic effects within a very short time.

In most clinical and experimental studies, HFOV is initiated with a LVOP with an initial CDP level 5 mbar above the mean airway pressure previously used in conventional ventilation [5,6,8,12]. The CDP is then increased in a stepwise fashion (2–5 mbar steps) every 15–30 minutes until a maximum in PaO_2 _or a predetermined CDP is reached. Our study presents a fast lung optimization procedure that could be technically applied easily in the clinical scenario. In contrast to recent studies in animals and in patients, the CDP was directly set to 45 mbar for 2.5 minutes. The lowest possible TPP that still assures the improved oxygenation is subsequently titrated.

Recruitment maneuvers are beneficial at the initiation of HFOV to ensure sufficient gas exchange area in the diseased lung [4]. In the Treatment with Oscillation and an Open Lung Strategy trial, the combination of HFOV and a high initial mean airway pressure recruitment maneuver without ongoing HFOV resulted in a rapid and sustained improvement in oxygenation [10]. Although sustained inflation pressures up to 55 mbar are necessary to overcome the opening pressure of collapsed alveoli [13], the criteria of a fully recruited lung, defined as pulmonary shunt proportion < 0.1 [14], was achieved at a CDP of 45 mbar. A CDP of 32 ± 6 mbar in piglets was able to reduce the shunt fraction < 10% [15]. During the present study a single pressure step-up for alveolar recruitment was performed, and therefore no estimate can be made of whether a lower CDP and TPP would have been adequate to reopen the lung, and whether the TPP required to open alveolar units exceeds the TPP required to maintain alveolar patency [15,16]. Assuming that the TPP stays above a critical closing pressure, a significant alveolar derecruitment cannot be expected [17,18]. In other experimental studies with the lavage-injury animal model, the mean airway pressures were set according to the pressure-volume curve for the setting of the CDP during HFOV [9,16,19]. In their study, Sedeek and colleagues repeated recruitment maneuvers with a continuous positive airway pressure of 50 mbar for 1 minute until the PaO_2 _was stable with a CDP set according to the maximal compliance on the pressure-volume curve [9].

We intended to use an alternative method without the pressure-volume curve. The P_a_O_2_/F_i_O_2 _ratio was therefore used as a criterion to identify the lowest TPP preventing alveolar derecruitment during the stepwise decrease of CDP. A TPP below this threshold was associated with an increased shunt > 10 and with an increased PaCO_2_. An increased PaCO_2 _at unchanged HFOV settings indicates a decreased alveolar surface available for gas exchange.

Previous studies in the lavage animal model showed that CO decreased at high mean airway pressures. Interestingly, better oxygenation values in those studies were found at lower mean airway pressure, suggesting in our study that eventually a lower CDP eventually would have been sufficient [16,19]. The lung recruitment maneuver had a marked effect on hemodynamics. These effects can easily be corrected by volume or by vasoactive drug usage. There was no relevant decrease of the intrathoracic blood volume as an indicator of reduced venous preload [20]. Also, the fluid regime before initiation of HFOV may have attenuated a reduction of venous return.

At a CDP of 45 mbar, the SV and CO were decreased and, simultaneously, the cardiac filling pressures (LVEDP, right atrial pressure, and PAOP) were elevated. This can be explained by a compression of the heart into the cardiac fossa due to the transmission of high TPPs [21,22]. Impairment of hemodynamics can therefore be explained by the mechanical restriction of the heart. Systemic afterload did not have a major impact on the impaired SV and CO as measured by the systemic vascular resistance. Right ventricular dysfunction may occur when high airway pressures are applied, and the consecutive right ventricular output and left ventricular filling are impaired leading to SV and CO decreases. As the pulmonary vascular resistance was unaffected by a CDP of 45 mbar and the right ventricle was able to generate a pressure gradient (mean pulmonary artery pressure–right atrial pressure), we excluded right ventricular failure. During the deceleration CDP trial, the cardiac filling pressures returned to similar values as measured before the lung recruitment maneuver. This observation assures us that the hemodynamic effects are related to a pressure transmission of the CDP and the TPP.

The safe window for plateau pressures during conventional ventilation is considered between 30 and 35 mbar, but even those values can lead to a harmful TPP. Depending on the elastance of the respiratory system, volutrauma may occur from cyclic tidal overdistension. No recommendations regarding safe levels for CDP or TPP exist during HFOV. The lowest TPP levels possible, however, should be applied. The measurement and monitoring of the TPP is therefore helpful and of increased interest, as the TPP is the effective distending force of the lung, and ventilator-induced lung injury (VILI) depends on the TPP [23,24]. By measuring the TPP the mechanical ventilator settings could be set more individually with respect to lung and chest wall mechanical characteristics, which enables the identification of lung recruitment potential in relation to a potential risk of VILI. Such an individual approach may reduce the risk for further lung injury in patients with ARDS undergoing mechanical ventilation [25,26]. In a clinical scenario, a CDP higher than the safe window for plateau pressures under conventional ventilation should be avoided even if suggested by the LVOP presented in this study. Although the theoretical advantage of HFOV is the avoidance of volutrauma caused by tidal overdistension (due to minimal tidal volumes) in the case of excessive TPP, a compromise between oxygenation and potential VILI should be made to avoid a VILI and to accept lower but adequate oxygenation.

The esophageal pressure measured by an esophageal balloon is used as a surrogate parameter of the pleural pressure for calculation of the TPP. The pleural pressure and therefore the TPP are dependent on the chest wall elastance in experimental patients and ARDS patients. For a given mean airway pressure (CDP) the pleural pressure increases if the chest wall elastance is elevated, and consequently the TPP decreases. Chest wall elastance, however, depends on the pathophysiology of ARDS (for example, high chest wall elastance in extrapulmonary ARDS). The elevated pleural pressure (due to increased chest wall elastance) leads to a lower TPP compared with pulmonary ARDS with normal elastance [1,27-29].

We found a pressure difference of about 10 mbar between the applied CDP and the resulting TPP. The difference in mean intrathoracic pressure and CDP in HFOV increases with the oscillatory frequency, decreasing the tracheal tube diameter and the relative duration of the inspiratory time [30]. The inspiration time:expiration time ratio of 33% in our study results in an increased tracheal tube resistance and, consequently, in a decreased mean intrathoracic pressure and TTP.

## Limitations

A major limitation of this study is that it was performed in a lavage animal model of ARDS and not in patients. Large-animal models, however, have contributed greatly to the understanding of the basic physiology of HFOV and its clinical application during severe lung injury [31]. ARDS in patients is rarely and solely a result of surfactant deficiency only. Early institution of HFOV, however, and institution of HFOV in patients with high potential for recruitment is theoretically beneficial to outcome in ARDS [32-34]. The lavage model is easily recruitable and is probably the most comparable experimental ARDS model for early-phase ARDS. Although it is primarily a model of surfactant depletion, it has been shown that mechanical ventilation after lavages leads to neutrophil infiltration, cytokine expression, and capillary-alveolar protein leak (as indicated by the increased extravascular lung water in this study) [35-37]. The present study only observed immediate changes of hemodynamics and lung oxygenation. High airway pressures and subsequent lung overdistension may lead to mediator release and to VILI. These adverse events were not accessible in the present study. Also, we cannot report whether the optimized gas exchange remained stable over a longer time period. This raises the question of how often such recruitment maneuvers should be applied in clinical practice.

## Conclusion

This short-term experimental observation study demonstrates the feasibility of a fast and safe lung optimization procedure during uninterrupted HFOV. This lung optimization procedure was well tolerated and resulted in a dramatic improvement of oxygenation. No clinically relevant adverse effects on hemodynamics or barotrauma occurred during the time period of approximately 1 hour. The stepwise decrease of CDP allowed the determination of the lowest TPP level necessary to maintain adequate oxygenation.

## Key messages

• A novel lung optimization procedure in uninterrupted HFOV is presented.

• The LVOP consists of a single CDP recruitment maneuver during uninterrupted HFOV, followed by a stepwise decrease of the CDP.

• This LVOP allows the determination of the lowest TPP that still maintains adequate pulmonary gas exchange.

• This LVOP shows no adverse effects in hemodynamics and oxygenation during its intervention in a porcine lavage ARDS model.

## Abbreviations

ARDS = acute respiratory distress syndrome; CDP = continuous distending pressure; CDP20 = continuous distending pressure of 20 mbar; CDP45 = continuous distending pressure of 45 mbar; CO = cardiac output; FiO_2 _= fraction of inspired oxygen; HFOV = high-frequency oscillatory ventilation; LVEDP = left ventricular end-diastolic pressure; LVOP = lung volume optimization procedure; MAP = mean arterial pressure; PaCO_2 _= arterial partial pressure of carbon dioxide; PaO_2 _= arterial partial pressure of oxygen; PAOP = pulmonary occlusion pressure; PCV = pressure-controlled ventilation; SV = stroke volume; TPP = transpulmonary pressure; VILI = ventilator-induced lung injury.

## Competing interests

The authors declare that they have no competing interests.

## Authors' contributions

JK designed the study protocol, collected data, and drafted the protocol. MD designed and participated in the study protocol, collected data, and performed statistical analysis. SY collected data. KM coordinated the study and revised the manuscript.

## Figures and Tables

**Table 1:** Hemodynamic data, blood gas data in pressure-controlled ventilation (PCV) pre-lavage, PCV post-lavage, and during lung volume optimization procedure in high-frequency oscillatory ventilation

	PCV pre-lavage	PCV post-lavage	45 mbar
Heart rate (beats/minute)	113 (112–124)	112 (102–125)	141 (126–167)
Right atrial pressure (mmHg)	9 (9–9)	10 (8–11)	16 (15–18)**
Mean pulmonary artery pressure (mmHg)	25 (20–37)	34 (30–41)	41 (35.7–52)**
Pulmonary occlusion pressure (mmHg)	10 (7–12)	13 (11–13)	24 (23–26)**
Left ventricular end-diastolic pressure (mmHg)	3 (1–4)	3.5 (2–6)	12 (11–13)**
Mean arterial pressure (mmHg)	89.5 (67–106)	83 (82–85)	85 (66–89)
Cardiac output (l/min)	3.28 (3.1–3.5)	3.6 (3.1–3.9)	2.6 (2.3–3.1)**
Stroke volume (ml)	31 (27–36)	32 (31–35)	19 (18–24)**
Intrathoracic blood volume (ml)	598.5 (548–622)	594 (553–690)	442 (401–469)
Extravascular lung water (ml)	290 (241–311)	420 (354–463)*	355 (315–476)
Systemic vascular resistance (dyn*s/cm^5^)	1817 (1277–2508)	1542 (1450–2074)	2403 (1524–2585)
Pulmonary vascular resistance (dyn*s/cm^5^)	266 (213–775)	545 (275–693)	639 (404–859)
Mean pulmonary artery pressure–right atrial pressure (mmHg)	16 (11–28)	26 (22–30)	28 (19–34)
Transpulmonary pressure (mbar)	4.5 (4–6)	10 (8–11)*	36 (32–43)**
PaO_2 _(Torr)	559 (535–658)	99 (56–128)*	621 (557–522)**
PaO_2_/FiO_2 _ratio	559 (535–658)	99 (56–128)*	621 (557–522)**
Oxygenation index	1.4 (1.2–2)	16.3 (14.6–21.3)*	7.2 (6.8–7.2)**
Oxygen delivery (ml O_2_/minute)	408.3 (382.5–427.6)	373 (297.6–427.9)	312 (299–466.2)
Shunt proportion	9.9 (8.8–11)	51.8 (49–55)*	1.03 (0.4–3)**
PaCO_2 _(Torr)	39.6 (32.8–48.4)	47 (40.7–58.8)	38.5 (28.3–46.6)
Oxygen saturation (%)	99.9 (99.9–99.9)	92 (89.7–96.5)*	99.9 (99–100)**
pH	7.45 (7.4–7.5)	7.38 (7.31–7.4)	7.41 (7.37–7.5)
Standard bicarbonate (mmol/l)	28.2 (27.8–30.2)	28.5 (26.2–30.9)	25 (23.2–27.5)
Base excess	4.8 (2.1–5.9)	2.25 (1–2.9)*	0.7 (-0.12 to 4.95)
Hemoglobin (g/dl)	7.6 (7.5–8)	7.8 (7.4–8.3)	7.9 (7.1–9.7)
Plateau airway pressure (cmH_2_0)	21 (10.8–14.4)	30 (25–35)	
Inspiratory tidal volume (ml)	335 (330–450)	390 (310–440)	
Dynamic lung compliance (ml/mbar)	12.6 (10.8–14.4)	7.9 (5.3–9.8)*	

Data presented as median (25th–75th percentiles). *P < 0.05 vs PCV pre-lavage (Wilcoxon signed-rank test), **P < 0.05 vs PCV post-lavage.

**Table 2:** Hemodynamic data and blood gas data on every continuous distending pressure in high-frequency oscillatory ventilation study protocol

	Descent continuous distending pressure trial					
	
	45 mbar	40 mbar	35 mbar	30 mbar	25 mbar	20 mbar
Heart rate (beats/minute)	141.5 (126–167)	135.5 (112–153)	128 (113–149)	126 (107–147)	115 (102–140)	116 (104.5–128.5)
Right atrial pressure (mmHg)	16 (15–18)	15 (14–15)	14.5 (13–15)	13.5 (12–15)	12.5 (11–13)	11.5 (10–13)^†,**,*^
Mean pulmonary artery pressure (mmHg)	41 (35.7–52)	39 (35–41)	39 (31–44)	41 (35–42)	39 (35–43)	38.5 (36–42)
Pulmonary occlusion pressure (mmHg)	24 (23–26)	21.5 (20–24)	20 (18–21)	17 (16.2–18.7)	14 (13.5–17)^†^	13.5 (12–15)^†,**^
Left ventricular end-diastolic pressure (mmHg)	11.5 (11–13)	9 (9–11)	9.5 (6–10)	7.5 (6–12)	7.5 (5–10)^†^	5 (4–6)^†^
Mean arterial pressure (mmHg)	85 (66–89)	70 (63–90)	74 (67–104)	74.5 (66–101)	78.5 (77–108)	84 (47–106)
Cardiac output (l/minute)	2.6 (2.3–3.1)	2.6 (2–3)	2.65 (2.28–3.36)	2.52 (2.2–3.6)	2.7 (2.2–4.2)	3.1 (1.8–4.8)
Stroke volume (ml)	19 (18–24)	21 (16.6–24.5)	23.6 (15–29)	23 (15–26.9)	18.2 (16–33)	18.7 (12.8–37)
Intrathoracic blood volume (ml)	442.5 (401–469)	395 (335–495)	485 (431–531)	506.5 (453–542)	552 (412–618)	540 (492–614)
Extravascular lung water (ml)	355 (315–476)	360 (331–441)	405 (287–430)	426 (336–495)	441 (406.5–535)	460 (390–549)
Systemic vascular resistance (dyn*s/cm^5^)	2,403 (1,524–2,585)	1,598 (1,582–2,271)	1,835 (1,567–1,997)	2,026 (1,442–2,033)	1,933 (1,303–2,469)	1,611 (1,238–2,056)
Pulmonary vascular resistance (dyn*s/cm^5^)	639 (404–859)	440 (396–663)	457 (376–663)	512 (461–746)	617 (418–970)	678 (415–1,065)
Mean pulmonary artery pressure–right atrial pressure (mmHg)	28 (19–34)	25.5 (23–26)	25 (18–29)	27.5 (26–28)	27 (25–30)	28.5 (23–31)
Transpulmonary pressure (mbar)	36 (32–43)	31 (27–38)	26.5 (22–33)	21.5 (17–28)	14.5 (12–22)^†,**^	10 (7–18)^†,**^
PaO_2 _(Torr)	621 (619–660)	654 (645–683)	664 (636–694)	644 629–692)	615 (469–680)	429 (52–558)**
PaO_2_/FiO_2 _ratio	82.8 (82.5–88)	654 (645–683)	664 (636–694)	644 629–692)	615 (469–680)	429 (52–558)**
Oxygenation index	7.2 (6.8–7.2)	6.1 (5.8–6.1)	5.4 (5–5.5)	4.6 (4.3–4.7)	4 (3.6–5.3)	4.8 (3.5–37.9)
Oxygen delivery (ml O_2_/minute)	312.5 (299–466)	358.2 (305.6–420)	360 (349–418)	357.2 (338–447)	347.4 (292–516)	399.6 (251–548)
Shunt proportion	1.03 (0.4–3)	0.7 (0.1–1.4)	1.5 (0–4.3)	1.6 (1.1–4.9)	4.7 (4.2–11.3)	20.2 (7.2–52)^†,**^
PaCO_2 _(Torr)	38.5 (28.3–46.6)	40.3 (30.1–51.1)	37.4 (30.7–44.8)	41.2 (31.1548)	46.9 (32.9–61.2)	54.5 (40.7–68.6)^†,*^
Oxygen saturation (%)	99.9 (99–100)	99.9 (99.8–100)	99.9 (99.9–100)	99.9 (98.3–100)	99.9 (96–100)	99.8 (83–99)
pH	7.41 (7.37–7.5)	7.4 (7.34–7.53)	7.4 (7.38–7.51)	7.4 (7.38–7.51)	7.38 (7.35–7.48)	7.29 (7.25–7.4)
Standard bicarbonate (mmol/l)	25 (24.2–29.8)	25.8 (25–27)	26 (23.4–27.6)	25.1 (23.5–27.7)	25.3 (24.2–28.5)	24.6 (24.1–28.5)
Base excess	0.95 (0.4–6.2)	1 (-1.5 to 5.2)	02 (-0.65 to 2.97)	-0.15 (-2.4 to 1.6)	-1.35 (-2.3 to 1.4)	0.2 (-4.25 to 0.47)
Hemoglobin (g/dl)	7.9 (7.1–9.7)	8.8 (7.4–10.1)	8.2 (7.5–10.4)	8 (7.4–9.7)	7.9 (7.1–9.4)	7.8 (7–10)

Data presented as median (25th–75th percentiles). Significant differences in parameters are indicated: *P < 0.05 vs 35 mbar, **P < 0.05 vs 40 mbar, ^†^*P *< 0.05 vs 45 mbar.
